# *Bacillus toyonensis* Strain AEMREG6, a Bacterium Isolated from South African Marine Environment Sediment Samples Produces a Glycoprotein Bioflocculant

**DOI:** 10.3390/molecules20035239

**Published:** 2015-03-23

**Authors:** Kunle Okaiyeto, Uchechukwu U. Nwodo, Leonard V. Mabinya, Anthony I. Okoh

**Affiliations:** 1 SA-MRC Microbial Water Quality Monitoring Centre, University of Fort Hare, Alice 5700, South Africa; E-Mails: UNwodo@ufh.ac.za (U.U.N.); LMabinya@ufh.ac.za (L.V.M.); AOkoh@ufh.ac.za (A.I.O.); 2 Applied and Environmental Microbiology Research Group, Department of Biochemistry and Microbiology, University of Fort Hare, Alice 5700, South Africa

**Keywords:** marine environment, *Bacillus toyonensis* strain AEMREG6, REG-6, flocculating activity, glycoprotein, thermostable

## Abstract

A bioflocculant-producing bacteria, isolated from sediment samples of a marine environment in the Eastern Cape Province of South Africa demonstrated a flocculating activity above 60% for kaolin clay suspension. Analysis of the 16S ribosomal deoxyribonucleic acid (rDNA) nucleotide sequence of the isolate in the GenBank database showed 99% similarity to *Bacillus toyonensis* strain BCT-7112 and it was deposited in the GenBank as *Bacillus toyonensis* strain AEMREG6 with accession number KP406731. The bacteria produced a bioflocculant (REG-6) optimally in the presence of glucose and NH_4_NO_3_ as the sole carbon and nitrogen source, respectively, initial medium pH of 5 and Ca^2+^ as the cation of choice. Chemical analysis showed that purified REG-6 was a glycoprotein mainly composed of polysaccharide (77.8%) and protein (11.5%). It was thermally stable and had strong flocculating activity against kaolin suspension over a wide range of pH values (3–11) with a relatively low dosage requirement of 0.1 mg/mL in the presence of Mn^2+^. Fourier transform infrared spectroscopy (FTIR) revealed the presence of hydroxyl, carboxyl and amide groups preferred for flocculation. Scanning electron microscopy (SEM) revealed that bridging was the main flocculation mechanism of REG-6. The outstanding flocculating performance of REG-6 holds great potential to replace the hazardous chemical flocculants currently used in water treatment.

## 1. Introduction

Industrial wastewater treatment is a hot research topic globally; flocculation has been recognised to be an excellent approach for removing pollutants from wastewater [1]. Although, many inorganic and organic synthetic flocculants exhibit good flocculation efficiency but their environmental and health problems cannot be neglected [2]. Alternatively, bioflocculants could be substitutes due to their innocuousness and biodegradability [3]. Bioflocculants are secondary metabolites produced during the growth of microorganisms, which are predominantly composed of polysaccharides, proteins, nucleic acids and lipids [4]. Amongst these macromolecules, the polysaccharide-based bioflocculants have attracted wide attention because of their high rates of flocculation in removing different kinds of heavy metals, cell removal and biomass recovery, and waste/drinking water treatment [5,6,7].

Several reports have shown the production of bioflocculants by bacteria, fungi, and algae isolated from activated sludge, soil and water [8,9,10]. Fermentation conditions and nutritive components of the cultivation medium have been reported to have a great influence on bioflocculant production [11]. Due to the low flocculating capability and high cost of production, industrial production of bioflocculants is not well established. Consequently, there is a need to seek microorganisms with greater bioflocculant production capability to reduce the production cost [8]. Furthermore, it will be economically auspicious to investigate technology for improving the flocculating activity of the purified bioflocculants.

From previous studies, species of the genus *Bacillus* isolated from freshwater sources are well documented to produce bioflocculants [12,13,14,15]; however, the production of bioflocculants by *Bacillus* species isolated from the marine environment is still scarce [16]. The marine environment is the largest habitat on the Earth, accounting for more than 90% of the total biosphere volume [17]. It is one of the most adverse environments due to the varying temperature, salinity and pH conditions. Besides, due to the constant variation of environmental conditions, the microorganisms present in that environment are suitably adapted to these adverse conditions since they exhibit complex adaptation features [18].

*Bacillus toyonensis* is a Gram-positive, spore-forming bacteria that forms a homogeneous independent branch within the *Bacillus* genus. It has a prodigious economic importance, for example the spores of *Bacillus toyonensis* BCT–7112 have been used in animal nutrition in some parts of the world [19]. In this paper, we report on bioflocculant production by *Bacillus toyonensis* strain AEMREG6 isolated from sediment samples from Algoa Bay in the Eastern Cape Province of South Africa. To the best of our knowledge, this is the first study to implicate the species *Bacillus toyonensis* in bioflocculant production.

## 2. Results and Discussion

### 2.1. Identification of the Bioflocculant-Producing Bacteria

The bacteria used in this study were isolated from sediment samples from Algoa Bay (a marine environment) in the Eastern Cape of Province South Africa screened for the ability to flocculate a kaolin clay suspension. The bacteria showed good bioflocculant production potential, having flocculating activity of over 60% for kaolin clay suspension. The bacterial colonies appear to be creamy, smooth and viscous on nutrient agar plate. The identity of the isolate was confirmed by 16S rDNA analysis. The BLAST analyses of the nucleotide sequence of the 16S rDNA of the bacterium showed a 99% similarity to *Bacillus toyonensis* strain BCT-7112, and the nucleotide sequence was deposited in the GenBank as *Bacillus toyonensis* strain AEMREG6 with accession number KP406731. Many *Bacillus* species have been implicated in bioflocculant production in previous studies [1,2,3,4,5,6,7,8,9,10,11,12,13,14,15,19,20,21,22,23], but nowhere in the literature is *Bacillus toyonensis* reported as a bioflocculant producer.

### 2.2. Optimization of Culture Conditions for REG-6 Production

#### 2.2.1. Effect of Carbon and Nitrogen Sources on REG-6 Production

**Figure 1:**
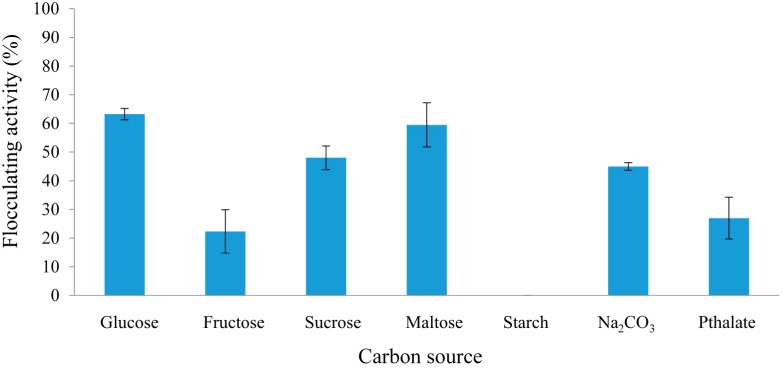
Effect of carbon source on REG-6 production by *Bacillus toyonensis* strain AEMREG6.

One of the most critical factors influencing bioflocculant production is the carbon source in the growth medium [24]. Carbon sources generally used in culture media for bioflocculant production have a direct impact on the production cost of bioflocculants, which limits the market potential of these products [25]. In this respect, the effect of various carbon and nitrogen sources on REG-6 production by the bacterium was assessed. Among these carbon sources, glucose and maltose containing media showed flocculating activity over 60%, which is a measure of REG-6 production (Figure 1).

Several studies in the literature reporting on bioflocculant production were in accordance with our present study. Most microorganisms utilized for bioflocculant production in the literature preferred glucose as the sole carbon source [25,26]. On the other hand, fructose- and phthalate-containing media resulted in poor flocculating activity. Only a few studies have documented the use of Na_2_CO_3_ as a carbon source for bioflocculant production [27]. Maltose was the carbon source of choice for *Solibacillus silvestris* [28], although starch inhibited the production of bioflocculant by the bacterium. On the contrary, *Aspergillus parasiticus* and *Bacillus licheniformis* preferred starch as the carbon source for bioflocculant production [29,30].

**Figure 2:**
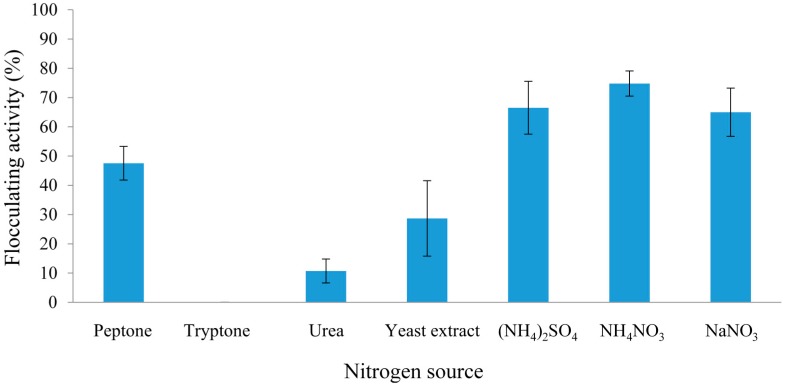
Effect of Nitrogen Source on REG-6 Production by *Bacillus toyonensis* strain AEMREG6.

The effect of different nitrogen sources on REG-6 production was examined and is presented in Figure 2. All the organic nitrogen sources tested were poorly utilized by this strain and resulted into low bioflocculant production. Comparatively, inorganic nitrogen sources greatly enhanced REG-6 production with the maximum flocculating activity of 73% being achieved with NH_4_NO_3_. Different inorganic nitrogen sources have been reported to be suitable for bioflocculant production in previous studies. For example, in a study documented by Nwodo *et al*. [26], (NH_4_)_2_SO_4_ was the most preferable nitrogen source for bioflocculant production among others.

In the same way, NH_4_Cl, (NH_4_)_2_SO_4_ and NaNO_3_ were the most favourable nitrogen sources for bioflocculant production by *Halomonas* sp., *Candida anglica* and *Gyrodinium impudicum* [30,31,32]. On the contrary, Li *et al*. [2] and Aijuboori *et al.* [33] found that peptone was most suitable for bioflocculants production by *Paenibacillus elgii* and *Aspergillus flavus*. Yeast extract was utilized for bioflocculants production by *Solibacillus silvestris*, *Kloeckera* sp. and *Penicillium purpurogenum* [28,34,35].

#### 2.2.2. Effect of Initial pH of Growth Medium on REG-6 Production

**Figure 3:**
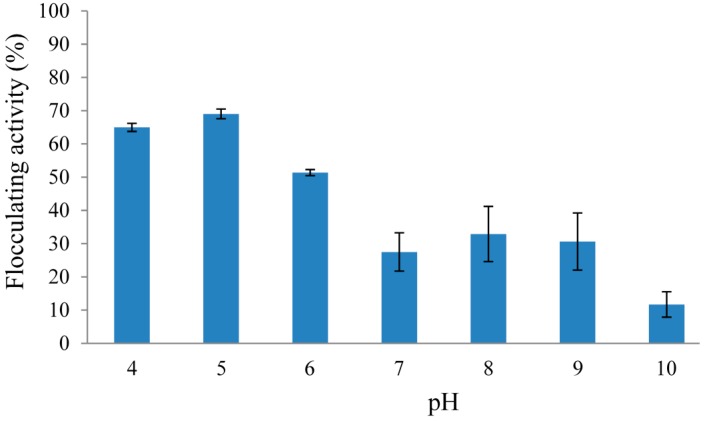
Effect of initial pH of growth medium on REG-6 production by *Bacillus toyonensis* strain AEMREG6.

It has been well documented in previous studies that the initial pH of the growth medium required for bioflocculant production differs between microorganisms [35,36]. According to Xia *et al.* [37], the initial pH of the growth medium affects the electric charge of the cells and redox reactions which in turn affect nutrient assimilation and enzymatic reaction. The effect of initial pH of the growth medium on REG-6 production was investigated in the pH range from 4 to 10 (Figure 3). It was observed that the bioflocculant production was better under acidic conditions, with the highest flocculating activity being 65% at pH 5. Further increases in pH either to neutral or alkaline conditions poorly supported the production of REG-6, with the lowest flocculating activity being observed at pH 10.

Similar findings were documented by Liu *et al.* [38] and Zufarzaana *et al.* [39] for bioflocculant production by *Chryseobacteria daeguense* W6 and *Bacillus* spp. UPMB13 under weak acidic condition to alkaline conditions. In the case of the bioflocculant produced by *Cobetia* sp., the maximum production was obtained at pH 6 [36]. On the contrary, the optimum pH for bioflocculant production by *Klebsiella* sp. TG-1 was pH 8 [3]. Likewise, an alkaline pH range of 7–12 was more suitable for bioflocculant production by *Bacillus* sp. F19, with the maximum flocculating activity being observed at pH 9, whereas acidic condition completely inhibited bioflocculant production [21].

#### 2.2.3. Effect of Inoculum Size on REG-6 Production

**Figure 4:**
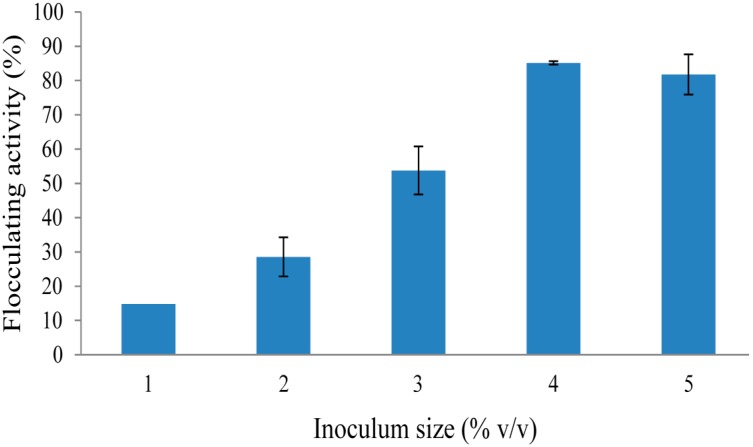
Effect of inoculum size on REG-6 production by *Bacillus toyonensis* strain AEMREG6.

Effect of inoculum size on REG-6 production by *Bacillus toyonensis* strain AEMREG6 was examined (Figure 4). Inoculum size play important role in cell growth and bioflocculant production. Small inoculum sizes tend to prolong the lag phase, while a large inoculum will make niches of the strain overlap excessively and consequently inhibit bioflocculant production [40]. In case of *Serratia ficaria* and *Aspergillus flavus*, optimum bioflocculant production was observed at 1 and 2% inoculum size, respectively [21,33]. In this present study, REG-6 production by the bacterium was increased with the increase in inoculum size, with the optimum production being observed at 4% inoculum size (Figure 4). Our findings were comparable to the report by Wang *et al.* [41] for bioflocculant production by *Klebsiella mobilis*, where the optimal production was observed at 5% inoculum size.

### 2.3. Optimization of the Flocculating Conditions of Crude REG-6

#### 2.3.1. Effect of Cations on the Flocculating Activity of Crude REG-6

Cations can neutralize the negative charges of both bioflocculant and suspended particles, and subsequently increase the adsorption of bioflocculant onto suspended particles [42]. As shown in Table 1, both monovalent and divalent cations enhanced the flocculating activity of produced REG-6 by more than 80%. The highest flocculating activity of 87% was observed with Ca^2+^, followed by K^+^ (85%), Mn^2+^ (84%), Mg^2+^ (82.2%), Na^+^ (81.3%) and Al^3+^ (71.3%). The flocculating activity of REG-6 was completely inhibited in the presence of Fe^3+^ due to excessive adsorption of the ions [42]. In another study reported by Nwodo *et al.* [26], the flocculating activity of the bioflocculant produced by *Brachybacteria* sp. was synergistically enhanced in the presence of Ca^2+^, Mg^2+^ and Mn^2+^.

**Table 1:** Effect of cations on the flocculating activity of crude REG-6 produced by *Bacillus toyonensis* strain AEMREG6.

Cations (w/v)	Flocculating Activity (%)
Li^+^	-
K^+^	-
Na^+^	-
Mg^2+^	86.82
Ca^2+^	84.96
Mn^2+^	89.5
Fe^3+^	-
Al^3+^	-

Likewise, Ca^2+^, Na^+^ and K^+^ were more effective in stimulating the flocculating activity of the bioflocculant produced by *Bacillus mojavensis* [14]. On the other hand, the addition of cations in a flocculation process usually increases treatment cost and besides, cations can also constitute environmental pollution. Hence, it will be propitious to produce a bioflocculant that is cation-independent. Some microorganisms such as *Solibacillus silvestris*, *Coryneobacteria daeguense* and *Citrobacter* sp.have been reported to produce such cation-independent bioflocculants [28,38,43].

#### 2.3.2. Effect of pH on the Flocculating Activity of Crude REG-6

**Figure 5:**
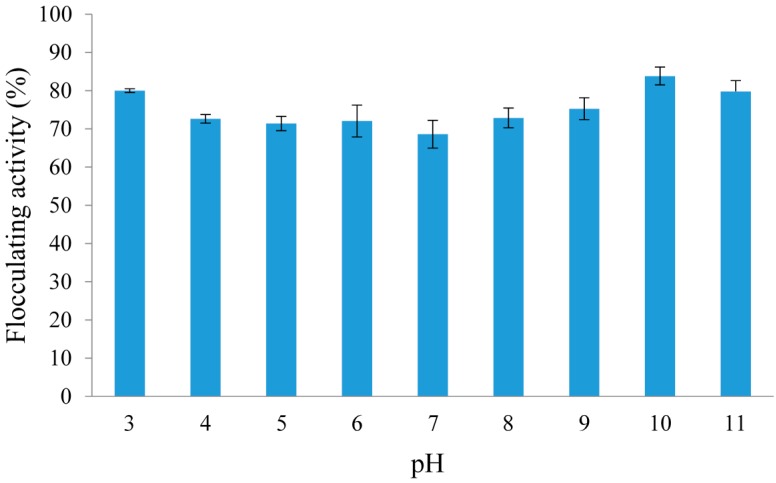
Effect of pH on the flocculating activity of crude REG-6 produced by *Bacillus toyonensis* strain AEMREG6.

The pH of the environment is one of the most important external factors influencing the flocculating activity of a bioflocculant [1]. Figure 5 shows the effect of pH on the flocculating activity of REG-6 as its exhibited different electrical charges at different pH value which in turn affect the bridging mechanism required for optimal flocculation [44]. REG-6 had high flocculating activity above 85% over a wide range of pH values from 3–11, with the maximum flocculating activity of 94% occurring at pH 3. Wang *et al.* [45] stated that pH determines the formation of flocs and also affects the stability of the suspended particles. The high flocculating activity shown over a wide pH range suggests that REG-6 can be applied under extreme environmental conditions which portends its good industrially applicability. Correspondingly, Zheng *et al*. [21] found that the best pH that supported flocculation of the bioflocculant produced by *Bacillus* sp. F19 was pH 2. In the case of the bioflocculant produced by *Ruditapes philippinarum*, it showed strong flocculating activity for kaolin clay suspensions over a wide pH range (1–13) with the optimum flocculating activity being observed in the range of 7–9 [46]. The bioflocculant produced by *Ochrobactrum ciceri* exhibited a good flocculating activity above 94% over the pH 1–10 range [28], whereas the bioflocculant produced by *Bacillus licheniformis* was relatively pH stable in the range of 2–11 [47]. When a bioflocculant only flocculates well or is stable in a narrow pH range, the possibility of its application under extreme conditions will be limited and the treatment cost will be high since there will be a need to adjust the pH of the water or wastewater to be treated to the desired pH range of the bioflocculant and consequently this inflates treatment costs.

### 2.4. Time Course of REG-6 Production by Bacillus Toyonensis Strain AEMREG6

**Figure 6:**
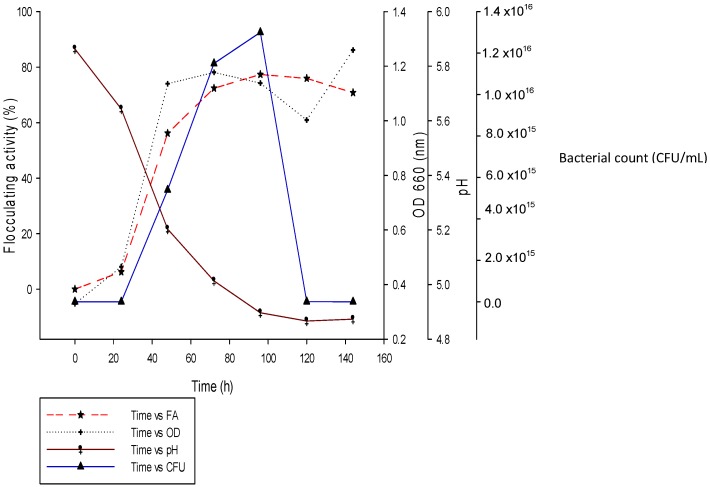
Time course of REG-6 production by *Bacillus toyonensis* strain AEMREG6.

The time course of REG-6 production by *Bacillus toyonensis* strain AEMREG6 was investigated and the results are presented in Figure 6. In previous studies, many researchers have reported that the production of bioflocculant was congruent with cell growth and maximum flocculating activity was achieved in the early stationary phase [48]. In Figure 6, the flocculating activity of the produced REG-6 increased progressively with the increase in cell growth, with the maximum flocculating activity of 77% being achieved after 96 h of cultivation. The cell growth reached a stationary growth phase between 48 and 96 h of cultivation after which the cell growth declined with a corresponding decrease in flocculating activity. This is an indication that bioflocculant production was associated with cell growth and not cell autolysis [8].

However, the decrease in flocculating activity observed after 96 h of cultivation could be due to the release of bioflocculant-degrading enzymes by the microorganism in the death phase of growth or the utilization of the produced bioflocculant as a carbon source for further cell growth [15,22]. Consequently, 96 h of cultivation was chosen for the subsequent experiments. We also observed a decrease in pH of the culture broth from 5.86 to 4.87 (Figure 6), and this decline in pH might be due to the production of organic acids from glucose or the presence of an organic acid as a component of REG-6 [49]. The growth pattern of *Aspergillus flavus* was observed to be closely-related to the flocculating activity of the produced bioflocculant [33].

Also, in the case of the bioflocculant produced by *Proteus mirabilis*, the flocculating activity also matched the cell growth pattern with the highest flocculating activity being observed in the stationary phase [37]. The bioflocculant production by *Bacillus mojavensis, Bacillus licheniformis* X-14 and *Klebsiella* sp. TG-1 reached a maximum after shorter cultivation times of 24, 20 and 28 h, respectively [14,22,25]. This indicated that these strains were able to produce bioflocculants at a lesser cultivation time than some other reported strains and consequently reduce the production cost.

### 2.5. Characterization of the Purified REG-6

#### 2.5.1. REG-6 Dosage

The effect of REG-6 dosage was investigated in the range of 0.05–0.5 mg/mL. The flocculating activity of the bioflocculant was highest at 0.1 mg/mL and further dosage increases resulted in a lower flocculating activity (Table 2). Lower dosages also induced lower flocculation efficiency of REG-6. The lower dosage effect implies that there were not enough bioflocculant molecules to adsorb the suspended kaolin clay particles to make the bridge process effective [50].

**Table 2:** Effect of dosage, pH, cations and temperature on the flocculating activity of purified REG-6.

Dosage (mg/mL)	FA (%)	pH	FA (%)	Cations	FA (%)	Temp (°C)	FA (%)
0.05	48.23	3	80	Li^+^	-	50	81.79
0.1	64.03	4	72.64	K^+^	-	60	81.87
0.2	54.29	5	71.41	Na^+^	-	70	82.74
0.3	55.37	6	72.05	Mg^2+^	86.82	80	81.94
0.4	46.79	7	68.61	Ca^2+^	84.96	-	-
0.5	45.51	8	72.86	Mn^2+^	89.51	-	-
-	-	9	75.29	Fe^3+^	-	-	-
-	-	10	83.84	Al^3+^	-	-	-
-	-	11	79.80	-	-	-	-

FA—Flocculating activity.

On the other hand, a higher dosage means the addition of the negatively charged REG-6 will increase the electrostatic repulsion forces between the kaolin clay particles; and consequently increase the distance between the kaolin clay particles which in turn inhibits floc formation and precipitation [51]. The relationship between bioflocculant dosage and flocculating activity of the purified bioflocculant was similar to that of the bioflocculants produced by other pure bacterial strains [14,32,38].

#### 2.5.2. Effect of pH on the Flocculating Activity of REG-6

The pH of reaction mixtures is a key factor influencing the flocculation activity of bioflocculants [15]. The effect of pH of the reaction mixture on the flocculating activity was investigated (Table 2). It was observed that REG-6 was tolerant to extreme pH values and showed excellent flocculating activity either under strongly acidic conditions below pH 5 or strongly alkaline conditions above pH 9. This may be due to the fact that REG-6 has different electric states at different pH values and this affects its flocculating efficiency for kaolin clay suspensions [52]. It was observed that the flocculating activity of REG-6 was highest under alkaline conditions (pH 10). Over 70% flocculating activity of the bioflocculant was observed at both strong acidic and alkaline conditions. In agreement with our findings, the bioflocculant produced by *Chlamydomonas reinhardtii* had its highest flocculating activity for kaolin clay suspensions at pH 10 [53]. Similarly, the bioflocculant MBF-5 showed an excellent flocculating activity (over 90%) under both acidic and alkaline conditions. The purified bioflocculant 40B produced by *Bacillus velezensis* 40B had its optimal flocculating activity under acidic conditions, with the peak flocculation occurring at pH 7 [15].

#### 2.5.3. Effect of Cations on the Flocculating Activity of REG-6

It is well-established that cations are necessary to induce effective flocculation by increasing the initial adsorption of the bioflocculant on the kaolin clay suspension [32]. The synergistic effects of cations on the flocculating activity of REG-6 are different from those of most other bioflocculants documented in the literature. In the present study, a synergistic effect of cations was only observed with the divalent cations Mg^2+^, Ca^2+^ and Mn^2+^, with the maximum flocculating activity of 89.51% being observed with Mn^2+^ (Table 2). These cations stimulated flocculation by accelerating bridge formation between the suspended particles and REG-6. The flocculating activity of REG-6 was completely inhibited by both the monovalent and trivalent cations tested.

On the contrary, the flocculating activity of the bioflocculant produced by *Aeromonas* sp. was greatly improved in the presence of K^+^, Na^+^ and Ca^2+^ [54]. In case of the bioflocculant produced by *Serratia ficaria*, the flocculating activity was greatly enhanced in the presence of Ca^2+^ and Mg^2+^, whereas Fe^3+^ and Al^3+^ inhibited the flocculating activity of the bioflocculant [55]. Bioflocculants reported by some researchers in previous studies are cation-independent. For example, the bioflocculants produced by *Klebsiella pneumoniae* and *Aspergillus flavus* showed outstanding flocculating activities for kaolin suspension in the absence of cations [7,33].

#### 2.5.4. Thermal Stability of REG-6

The thermal stability of the bioflocculant was tested at different temperatures for 60 min. It was observed that the bioflocculant was heat stable as it retained and maintained a high flocculating activity of 81.94% at 80 °C (Table 2). The heat stability of REG-6 is consistent with the general understanding that bioflocculants rich in polysaccharides are more resistant to heat than those that are mainly composed of proteins or have a lesser polysaccharide content [56]. Similar findings were documented by Liu *et al.* [38] and Gong *et al.* [55] for the bioflocculants produced by *Corynebacteria daeguense* and *Serratia ficaria*, respectively.

#### 2.5.5. REG-6 Yield and Chemical Analysis

About 3.2 g of purified REG-6 was obtained from 1 L of fermentation broth of *Bacillus toyonensis* strain AEMREG6 and this was higher than the bioflocculant yields reported elsewhere [54,55,56,57]. The chemical composition analysis revealed that purified REG-6 was a glycoprotein composed of polysaccharide (77.8%) and protein (11.5%). This shows that REG-6 has a polysaccharide as the main backbone in its molecular chain and this account for its high thermal stability. These results concur with previous studies on the bioflocculants produced by *Klebsiella pneumonia*, *Solibacillus silvestris* and *Bacillus agaradhaeren* C9 which are also thermally stable [7,28,58].

#### 2.5.6. Functional Group Analysis by FTIR

**Figure 7:**
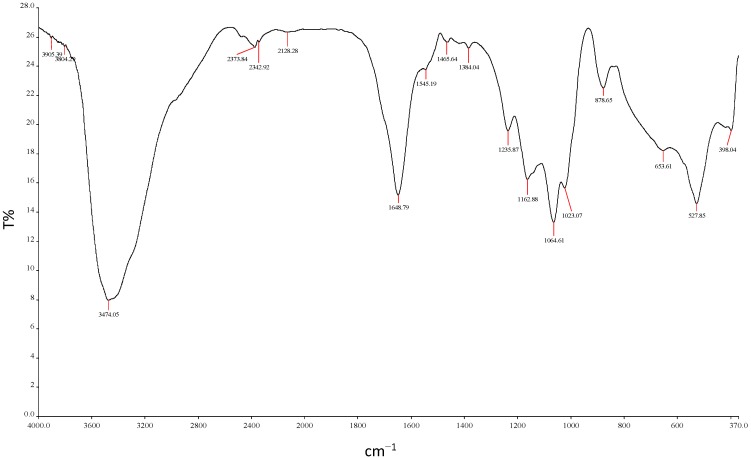
Functional groups identification by FTIR of REG-6 produced by *Bacillus toyonensis* strain AEMREG6.

The analysis of the functional groups of purified REG-6 produced by *Bacillus toyonensis* strain AEMREG6 showed a broad stretching peak at 3474 cm^−1^ (Figure 7), which is a common characteristic of hydroxyl and amino groups [22]. The spectrum also displays an asymmetrical stretching band at 1643 cm^−1^ which is consistent with the presence of carboxylates. Furthermore, the peak at 1465 cm^−1^ indicated the presence of carboxylic acid groups, and polysaccharide C-O and C–O–C groups. The strong absorption peak present in the range from 1000–1200 cm^−1^ is characteristic of all sugar moieties [56]. The small absorption band at 878 cm^−1^ could be associated with β-glycosidic linkages between the sugar monomers [59]. The peaks at 653 and 527 cm^−1^ are the absorption peaks for the aromatic CH bending vibration [60]. The FTIR analysis thus revealed the presence of carboxyl, hydroxyl and amide functional groups which might be responsible for the flocculation in polyelectrolytes [50]. The infrared spectra of REG-6 produced by *Bacillus toyonensis* strain AEMREG6 were similar to those of most bioflocculants produced by many microorganisms in previous studies [59,60,61,62].

#### 2.5.7. Scanning Electron Microscopy (SEM) Images

**Figure 8:**
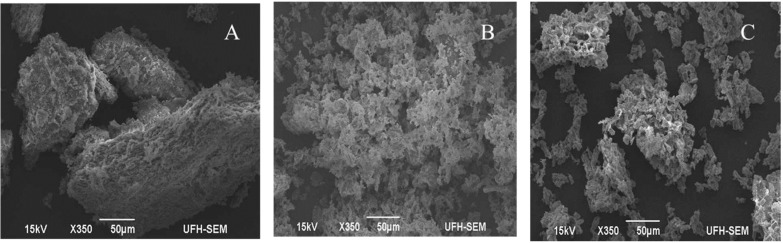
SEM images of REG-6 (**A**); kaolin clay particles (**B**) and kaolin clay suspension flocculated with REG-6 (**C**).

The surface morphology of purified REG-6 and its flocculation to kaolin clay suspension were examined by SEM. Figure 8A shows the compact nature of the REG-6 structure and Figure 8B shows the fine and scattered kaolin clay particles before flocculation. It was also observed that the sizes of the kaolin particles are uniform. Figure 8C can easily be compared with Figures 8A and B in terms of structure and sizes. It was observed that REG-6 flocculated the kaolin clay particles by connecting the scattered kaolin particles firmly together to form bigger flocs which easily precipitated as a result of gravity. These observations were consistent with the findings of Zhang *et al*. [50] and Mabrouk [63] on the bioflocculants produced by *Proteus mirabilis* and *Nocardiopsis aegyptia* sp. nov. respectively.

## 3. Experimental Section

### 3.1. Sample Collection and Isolation of Bioflocculant-Producing Bacteria

A sediment sample from Algoa Bay (a marine environment) in the Eastern Cape Province of South Africa was collected and processed according to Jensen *et al*. [64] with some modifications. A wet sample (0.5 g) was diluted with sterile seawater (5 mL). The suspension was stirred and allowed to sediment for 60 s, out of which 100 µL of the suspension was inoculated onto the surface of R2A agar plates, spread with a sterile glass rod and incubated for 96 h. The distinct isolates were picked and streaked onto nutrient agar plates to ascertain their purity and separate mixed populations.

### 3.2. Media and Cultivation Conditions

The composition of activation medium included (per litre): Beef extract 3 g, tryptone 10 g, NaCl 5 g [65]. Screening medium contained (per litre): glucose 20 g, K_2_HPO_4_ 5 g, KH_2_PO_4_ 2 g, (NH_4_)_2_SO_4_ 0.3 g, urea 0.5 g, yeast extract 0.5 g, MgSO_4_ 0.3 g, NaCl 0.1 g [66]. The medium for a slant included (per litre): glucose 20 g, K_2_HPO_4_ 5 g, KH_2_PO_4_ 2 g, (NH_4_)_2_SO_4_ 0.3 g, urea 0.5 g, yeast extract 0.5 g, MgSO_4_·7H_2_O 0.3 g, NaCl 0.1 g and agar 20 g [56]. Production medium composition was (per litre): glucose 20 g, K_2_HPO_4_ 5 g, KH_2_PO_4_ 2 g, NH_4_NO_3_ 1.3 g, MgSO_4_·7H_2_O 0.3 g, NaCl 0.1 g. All media were prepared using filtered seawater and sterilized by autoclaving at 121 °C for 15 min. In addition, all experiments were performed using a rotary shaker at 160 rpm, 28 °C.

### 3.3. Screening of Bioflocculant-Producing Bacteria

About 48 bacterial isolates were obtained from the Algoa Bay sediment sample and screened for bioflocculant production as follows. Two loopfulls of bacterial isolates from a nutrient agar plate were inoculated into activation medium and incubated for 24 h. One millilitre of the seed culture was inoculated into a 250 mL flask containing 50 mL of production medium and incubated at 28 °C in a rotary shaker at 160 rpm for 96 h. Two millilitres of the fermented broth were carefully withdrawn and centrifuged at 4000 rpm for 30 min, and the cell-free supernatant was next used to determine the flocculating activity according to the description of Kurane *et al*. [67]. The isolate with the highest flocculating activity (REG-6) was preserved in 20% glycerol stock and stored at −80 °C for future studies.

### 3.4. Determination of Flocculating Activity of REG-6

The flocculating activity of REG-6 was determined according to the description of Kurane *et al*. [67]. Kaolin clay was used as a test material in preparing a kaolin clay suspension which is a simulation of surface water turbidity. One hundred millilitres of the kaolin suspension (4 g/L) were measured into a 250 mL conical flask and 3 mL of CaCl_2_ (1% w/v) were added, followed by 2 mL of cell-free supernatant obtained by centrifuging a fermented culture after 96 h of cultivation. The solution was agitated for 60 s, transferred into a graduated measuring cylinder and allowed to sediment for 5 min. A control was prepared in a similar way, but the bioflocculant was replaced with un-inoculated production medium. The flocculating activity was calculated using the formula:

Flocculating activity (%) = [A − B/A] × 100

where A = optical density of the control at 550 nm and B = optical density of a sample at 550 nm.

### 3.5. Identification of the Bioflocculant-Producing Bacteria

The pure culture of the isolate was streaked on nutrient agar and incubated for 24 h. The purity of the isolate was ascertained and the isolate was then identified by 16S ribosomal deoxyribonucleic acid (rDNA) sequence analysis. DNA extraction was conducted using the boiling method described by Cosa *et al*. [57] whereby two to three colonies were suspended in 70 µL of sterile double distilled water. The samples were heated in a water bath at 100 °C for 10 min, cooled for 5 min and centrifuged at 3000 rpm for 5 min. The supernatant was transferred to a clean tube and stored at 4 °C. This serves as the template in the polymerase chain reaction (PCR) assay. PCR of the 16S rDNA was conducted according to the description of Zheng *et al*. [21] with some modifications using the universal primers (F1: 59-AGAGTTTGATCITGGCTCAG-39; I = inosine and primer R5: 59-ACGGITACCTTGTTACGA CTT-39) and 2 µL template DNA. Gel electrophoresis of PCR products was conducted on 1% agarose gels to confirm that a fragment of the correct size had been amplified. The PCR product was sequenced at University of KwaZulu-Natal Province (Durban, South Africa) and the results obtained were aligned with published 16S rDNA sequences in the GenBank through a BLAST sequence tool from the National Centre for Biotechnology Information (NCBI) database (Bethesda, MD, USA).

### 3.6. Optimization of Culture Conditions for REG-6 Production

#### 3.6.1. Effect of Carbon and Nitrogen Sources on REG-6 Production

The experiment to investigate the effect of carbon source on REG-6 production was done according to the description of Luo *et al*. [51], where glucose in the screening medium was replaced with 20 g/L of each of the following carbon sources: fructose, sucrose, maltose, Na_2_CO_3_ and phthalate. Similarly, the mixed nitrogen source (urea + yeast extract + (NH_4_)_2_SO_4_) in the screening medium was also replaced with 1.3 g/L of one of the following nitrogen sources: peptone, tryptone, urea, yeast extract, (NH_4_)_2_SO_4_, NH_4_NO_3_, and NaNO_3_ in order to examine the effect of nitrogen source on REG-6 production [68].

#### 3.6.2. Effect of Initial pH of Growth Medium on REG-6 Production

To evaluate the effect of initial pH of growth medium on REG-6 production, the pH of the media were adjusted to 3, 4, 5, 6, 7, 8, 9, 10 and 11 with 0.1 M HCl and NaOH accordingly. The seed culture (24 h old) was inoculated into production media at different pH values and incubated in a rotary shaker at 28 °C for 96 h at 160 rpm [69].

#### 3.6.3. Effect of Inoculum Size on REG-6 Production

To examine the effect of inoculum size on bioflocculant production, the seed culture (24 h old) was standardized to 0.1 at an optical density 660 nm, and then different inoculum sizes ranging from 1% to 5% were inoculated into different flasks containing the production medium and incubated for 96 h [66].

### 3.7. Optimization of Flocculating Conditions of Crude REG-6

#### 3.7.1. Effect of Cations on Flocculating Activity of Crude REG-6

To assess the synergistic effect of various cations on the flocculating activity of REG-6, the CaCl_2_ in the flocculation assay described in Section 3.4 was replaced with the metals of the following salts: KCl, NaCl, MgCl_2_, MnCl_2_, FeCl_3_ and AlCl_3_ [15].

#### 3.7.2. Effect of pH on the Flocculating Activity of Crude REG-6

To investigate the effect of pH on the flocculating activity of crude REG-6, 100 mL of kaolin clay suspension was measured into a 250 mL conical flask and the pH was adjusted from 3 to11 with 0.1 M HCl and NaOH [37]. The flocculating activity of REG-6 at different pH values was determined by the flocculation assay described above.

### 3.8. Time Course of REG-6 Production by Bacillus Toyonensis Strain AEMREG6

The time course of REG-6 production was conducted according to the description of Yang *et al.* [70] with some modifications. The seed culture (24 h old) was standardized to OD_660_ 0.1, then 4% v/v of standardized culture was inoculated into 1 L of production medium and incubated in a rotary shaker at 28 °C, 160 rpm for 96 h. The fermentation broth was withdrawn periodically and monitored at 24 h intervals, while the bacterial counts were determined by the culture technique to determine the colony forming units. Likewise, the optical density of the fermentation medium was measured spectrophotometrically (Helios Epsilon, Madison, WI, USA) at 660 nm wavelength and pH of the fermented broth was measured with pH meter.

### 3.9. Extraction and Purification of REG-6

The extraction and purification of REG-6 was done according to the Wong *et al.* [71]. After 96 h of fermentation, the viscous culture broth was diluted with two volumes of distilled water and centrifuged at 4000 rpm for 30 min at 15 °C in order to remove the bacterial cells. Two volumes of ethanol were added to the supernatant to precipitate REG-6. The resulting precipitate was collected by centrifugation at 4000 rpm for 30 min, dissolved in water and lyophilised to obtain the crude REG-6. The bioflocculant was dissolved in 100 mL of distilled water to which one volume of a mixture of chloroform and *n*-butyl alcohol (5:2 v/v) was added, stirred for 60 s and kept overnight at room temperature. The upper phase was collected by centrifugation at 4000 rpm for 30 min at 15 °C, re-dissolved in 50 mL of distilled water and lyophilised.

### 3.10. Characterization of the Purified REG-6

#### 3.10.1. Chemical Composition Analysis of REG-6

The total sugar content of REG-6 was determined according to phenol-sulphuric acid method described by Chaplin and Kennedy [72] using glucose as the standard. The total protein contain was determined by the Bradford method described by Bradford [73] using bovine serum albumin (BSA) as the standard.

#### 3.10.2. Effect of REG-6 Dosage on Flocculating Activity

To find a suitable dosage for the flocculating activity of purified REG-6, different dosages ranging from 0.1 to 0.5 mg/mL were prepared in distilled water and their flocculating activities were examined subsequently.

#### 3.10.3. Effect of pH on the Flocculating Activity of REG-6

The experiments concerning the effect of pH on the flocculating activity of purified REG-6 were carried out by adjusting the pH of the kaolin clay suspension from pH 3–11 with 0.1 M HCl and NaOH as needed [38]. The purpose of using a wide range of pH values is to determine the optimal pH range at which the flocculating activity of REG-6 will be optimum.

#### 3.10.4. Effect of Cations on the Flocculating Activity of REG-6

The synergistic effect of various cations on the flocculating activity of REG-6 was examined by replacing CaCl_2_ in the flocculation assay with metal of the following salts; KCl, NaCl, MgCl_2_, CaCl_2_, MnCl_2_, FeCl_3_ and AlCl_3_ [7]. The pH of the kaolin clay suspension was adjusted to pH 10 prior to flocculation assay.

#### 3.10.5. Thermal Stability of REG-6

The thermal stability was investigated by preparing REG-6 solution in distilled water at 0.1 mg/mL. The solution was divided into four groups and treated at different temperatures ranging from 50 to 80 °C [59].

#### 3.10.6. FTIR Analysis of REG-6

The functional groups in the molecular chain of REG-6 were identified using a Fourier transform infrared spectrophotometer (Perkin Elmer System 2000, Buckinghamshire, UK). REG-6 was ground with KBr salt at 25 °C and pressed into a pellet for FTIR spectroscopy analysis over a wavenumber range of 4000–370 cm^−1^ [13].

#### 3.10.7. Scanning Electron Microscopy (SEM) Images

The surface morphology of REG-6 and kaolin clay particles was observed and elucidated using scanning electron microscopy (SEM) (JSM-6390LV, JEOL, Tokyo, Japan). Five milligrams of REG-6, kaolin clay and dried flocculated kaolin suspension were added on slide separately and fixed by air-drying. The fixed specimens were coated with gold and examined under SEM [74].

### 3.11. Statistical Analysis

All data were obtained in triplicate experimentation and subjected to one-way analysis of variance (ANOVA) using the MINITAB Student Release 12 statistical package. A significance level of *p* ˂ 0.05 was used.

## 4. Conclusions

Owing to the stupendous advantages such as innocuousness and biodegradability of bioflocculants over chemical flocculants, the study of microbial flocculants has attracted wide attention in the water treatment field. In this present study, the bioflocculant REG-6 was optimally produced when glucose, NH_4_NO_3_, calcium chloride and pH 5 as favourable carbon, nitrogen source, cation of choice and initial growth medium pH were used, respectively. The high flocculating efficiency achieved at low dosage over a wide pH range and its thermal stability properties show that REG-6 has enormous potential for becoming a new member of the bioflocculants isolated and studied by our research group so far from Algoa Bay in the Eastern Cape Province of South Africa. Consequently, it is anticipated that the REG-6 produced by *Bacillus toyonensis* strain AEMREG6 could be a good substitute for the hazardous chemical flocculants currently used in drinking/wastewater treatment.
